# Reconstructing interpretable features in computational super-resolution microscopy via regularized latent search

**DOI:** 10.1017/S2633903X24000084

**Published:** 2024-05-30

**Authors:** Marzieh Gheisari, Auguste Genovesio

**Affiliations:** Institut de Biologie de l’Ecole Normale Supérieure (ENS), PSL Research University, Paris, France

**Keywords:** diagnostic, generative prior, microscopy, super-resolution

## Abstract

Supervised deep learning approaches can artificially increase the resolution of microscopy images by learning a mapping between two image resolutions or modalities. However, such methods often require a large set of hard-to-get low-res/high-res image pairs and produce synthetic images with a moderate increase in resolution. Conversely, recent methods based on generative adversarial network (GAN) latent search offered a drastic increase in resolution without the need of paired images. However, they offer limited reconstruction of the high-resolution (HR) image interpretable features. Here, we propose a robust super-resolution (SR) method based on regularized latent search (RLS) that offers an actionable balance between fidelity to the ground truth (GT) and realism of the recovered image given a distribution prior. The latter allows to split the analysis of a low-resolution (LR) image into a computational SR task performed by deep learning followed by a quantification task performed by a handcrafted algorithm based on interpretable biological features. This two-step process holds potential for various applications such as diagnostics on mobile devices, where the main aim is not to recover the HR details of a specific sample but rather to obtain HR images that preserve explainable and quantifiable differences between conditions.

## Impact Statement

This research addresses a crucial challenge in single-image super-resolution (SISR) by introducing Regularized Latent Search (RLS). Traditional SISR models depend on large paired datasets and offer only moderate resolution enhancements. Our method overcomes these limitations by leveraging the latent space of pre-trained generative models to find plausible HR images corresponding to LR inputs. This approach not only removes the necessity for paired images but also allows for a significant resolution boost of up to 32x. The significance of our approach lies in its ability to balance fidelity to the ground truth and realism of the recovered image given a distribution prior. By preserving biologically relevant content and interpretable features, our method ensures that super-resolved images are not only faithful to the original LR images but also retain essential domain-specific characteristics. This balance between fidelity and realism is crucial for accurate phenotypic interpretation and quantitative analysis, making our method particularly valuable for medical diagnostics and biological research.

## 1. Introduction

Various deep learning models have shown excellent performance in the single-image super-resolution (SISR) task, which aims to restore a high-resolution (HR) image from its low-resolution (LR) counterpart. Deep learning SISR models have been applied few years ago to enhance the resolution of microscopy images.^(^^1^^–^^3^^)^ More recently, studies have explored image-to-image translation models, which are trained to learn a parameterized function between two different image resolutions or modalities. These supervised approaches require a large number of paired images and rely on generative models that output artificial images.^(^^4^ ^)^ These artificial images were accepted by the community as real mainly because they were measured as approaching real image instances. However, for the latter to hold, these approaches only offered a moderate increase in resolution of up to 4x.

Importantly, learning a parameterized mapping from a LR image to a HR image is an ill-posed problem: A single LR image corresponds to infinitely many highly resolved ones. Therefore, the super-resolution (SR) task cannot only consist of maximizing the fidelity of the recovered HR image; it must be further constrained to be well-posed. In this work, we propose to constrain this task by imposing that the reconstructed SR image be realistic, *that is*, belongs to a given image distribution, a principle that is also explored by utilizing gradient distribution prior in the context of biomedical images.^(^^5^ ^)^ In short, on the one hand, fidelity refers to recovering a SR image that, once downgraded, is close to the original image. On the other hand, realism refers to keeping the image within a given image domain.

In this paper, we aim to better define the SR task by enforcing the solution to be a trade-off between fidelity to the original LR image and realism, which includes the preservation of biologically relevant content, which we describe as “interpretable features.” Assessing these features is essential for accurate phenotypic interpretation and discrimination. By guiding the SR process toward this end, we generate images that are both faithful to the sample and biologically interpretable and quantifiable. In this way, we anticipate that SR in biology could exploit more than recovering only the details of a given sample, but benefit from features measured over a set of recovered images.

Our approach, regularized latent search (RLS), consists of a regularized search in the latent space of a pretrained generative model for HR images. By doing so, we both suppress the need for paired images and make the problem well-posed as we search for the closest image a generator can produce that, when downscaled, matches the LR image input. Moreover, as we keep the super-resolved image in the original domain, instead of producing a moderate increase in resolution, we propose to push further the synthesis: We anticipate that creating very highly resolved (up to 32x) but controlled artificial images could be of great interest for applications such as diagnostic. This is because what is at stake, in this case, is the preservation of measurable and interpretable features of microscopy images, not the absolute matching with real image samples.

Similar to image-to-image translation methods like CycleGAN,^(^^6^ ^)^ which operate without the need for paired images, our method differentiates itself in its approach to SR. It is specifically designed to address the ill-posed nature of SR by utilizing a generative adversarial network (GAN)’s latent space to identify plausible HR images for any given LR input. Once the model is trained, it enables the SR of any LR image with just an adjustment in the degradation function during inference. Unlike CycleGAN, which is deterministic and tailored for specific domain translations requiring individual models for each task, our method offers versatility and is suitable for a broad spectrum of SR tasks. It eliminates the need to learn domain-specific features and is intended for widespread application in SR, capable of being trained on a single dataset to enhance any LR image.

## 2. Related work and background

### 2.1. SR of microscopy images

While optical SR techniques such as stimulated emission depletion (STED)^(^^7^ ^)^ and photoactivation localization microscopy (PALM)^(^^8^ ^)^ can break the diffraction limit, they are limited by the need for specialized equipment and complex sample preparation. Computational SR methods, on the other hand, while they rely on existing training data, can represent a cheap way to enhance the resolution of images acquired with conventional microscopes. Several approaches were developed to address the problem of computational SR of microscopy images. Deep-STORM (Stochastic Optical Reconstruction Microscopy)^(^^9^ ^)^ uses an encoder–decoder network to localize emitters in super-resolved images. Content-aware image restoration^(^^1^ ^)^ uses a U-Net architecture and is trained with low signal-to-noise ratio (input) and high signal-to-noise ratio (target) image pairs. These approaches produced interesting results, but recovered images often lacked high-frequency details due to the mean squared error (MSE) loss. To balance this issue, other approaches such as ANNA-PALM (Artificial Neural Network-based Photo Activated Localization Microscopy)^(^^3^ ^)^ are based on GAN. The authors trained a U-Net, and in contrast with Deep-STORM, a combination of pixel-wise reconstruction loss and adversarial losses is used to obtain image reconstructions of better quality. Using a similar network architecture and training loss, Wang et al.^(^^2^ ^)^ achieved SR in fluorescence microscopy across different modalities. Overall, using an adversarial loss results in output images that are sharper and of better perceptual quality and these methods demonstrated the potential of deep learning to improve the spatial resolution of fluorescence microscopy images. While the deep learning architectures and applications of these methods differ, they all require hard-to-get paired image data for training and offer a moderate increase in resolution of up to 4x.

It is essential to note that computational SR techniques effectively enhance the resolution of images by optimizing the use of available data in the original images. However, these methods are constrained by the existing information and cannot add details beyond what the original optical systems could capture.

### 2.2. Style-based generative models

StyleGAN (Style-based Generative Adversarial Network) models^(^^10^ ^,^^11^ ^)^ are well known for their ability to generate highly realistic images. The StyleGAN architecture consists of two subnetworks: a mapping network denoted by 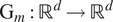 and a synthesis network consisting of 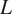 layers denoted by 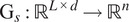 . Here, 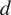 represents the dimensionality of the latent space. The mapping network takes a sample 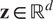 from a standard normal distribution and maps it to a vector 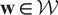 , where 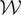 denotes the intermediate latent space. StyleGAN2 introduced path length penalty, which encourages a fixed-size step in 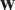 to result in a fixed-magnitude change in the image. The regularization term is computed using the Jacobian determinant, and penalizing changes in it promotes the generation of smoother and more realistic images. 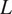 copies of the *d*-dimensional vector 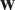 are fed to the 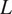 -layer synthesis network 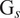 , with each copy representing the input to the corresponding layer of 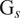 . The 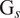 network controls the level of detail in the generated image at each layer. Individual modification of these 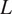 layers, by adjusting the latent vector copy of 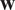 for each layer, extends the latent space into 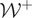 . This extended latent space enhances the model’s capability for accurate image reconstruction, which is vital for SR tasks, offering more nuanced control over the image generation process.^(^^12^ ^,^^13^ ^)^

### 2.3. GAN-based high-resolution image reconstruction

The problem of obtaining a SR image of dimension 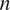 from a low-resolution (LR) image of dimension 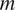 is ill-posed as, for a non-invertible forward operator 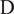 with 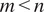 , there are infinitely many HR images that match a given LR image. Thus, the reconstruction procedure must be further constrained by prior information to better define the objective and lead to a stable solution. One such prior consisted of considering the reconstructed HR image to be part of a given domain. First, the distribution of HR images is learned in an unsupervised fashion, thanks to a GAN, and then the latent space of this trained GAN is searched to find a latent vector producing an HR image that, once downscaled, is the closest to the LR image input. GAN prior-based image reconstruction was first introduced by Bora et al.^(^^14^ ^)^ and further improved using StyleGAN^(^^10^ ^,^^11^ ^)^ by Menon et al. in PULSE (Photo Upsampling via Latent Space Exploration)^(^^15^ ^)^ by constraining the search to remain on the image manifold. To this end, PULSE and two other studies^(^^13^ ^,^^16^ ^)^ use an invertible transformation of the intermediate latent space 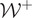 _,_ which includes a leaky rectified linear unit (rectified linear unit (ReLU))^(^^17^ ^)^ followed by an affine whitening transformation, so that transformed latent vectors approximately followed the standard Gaussian distribution 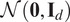 . Sampled vectors are then constrained to lie around a hypersphere with radius 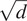 hypothesizing that most of the mass of a high-dimensional Gaussian distribution is located at or near 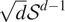 , where 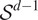 is the 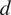 -dimensional unit hypersphere. Constraining samples to lie in a dense area of the StyleGAN style distribution resulted in increased realism of the generated images.

Although the above approach showed major improvements over previous work, it also presents important caveats that in practice led to image artifacts. Here, we show that transforming the intermediate latent space in this way does not lead to an accurate standard Gaussian distribution, and so prevents proper regularization based on this hypothesis. Moreover, as the search is strictly limited to the spherical surface 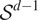 , this limitation may prevent the search from reaching the closest reconstruction of the HR image.

In this work, we regularize the search in the latent space for a latent code located in “healthy” regions of the latent space. In this way, the system is constrained to produce images that belong to the original image domain StyleGAN was trained on. To do so, we take advantage of normalizing flow to Gaussianize the latent style sample distribution, which leads to a much closer standard Gaussian distribution. We then use this revertible transformation to regularize the search in 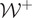 such that it remains in a high-density area of the style vector distribution. We then show experimentally that the latter produces reconstructed images that are not only realistic but also more faithful to the original HR image.

## 3. Method

### 3.1. Super-resolution by Regularized Latent Search

SR aims to reconstruct an unknown HR image 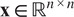 from a LR image 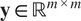 , which is related to the HR image by a downscaling process described by 
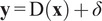
 , where 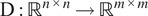 is a non-invertible downscaling forward operator and 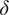 is an independent noise with distribution 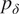 . We can formulate this task in terms of maximum a posteriori (MAP) estimation.^(^^18^ ^)^ Given an LR image 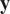 , our goal is to recover the HR image 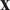 as the MAP estimate of the conditional distribution 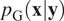 :(1)

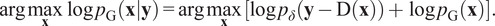



The first term is the likelihood term, describing the image degradation process 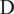 _,_ and the second is the image prior, describing the manifold of real HR images.

#### 3.1.1. Image prior

Let 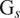 be the synthesis network of a StyleGAN^(^^11^ ^)^ pretrained on the considered image domain. 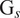 takes as input 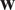 , produced by the mapping network, and outputs an image. The image prior 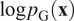 can be expressed with respect to the latent variables 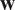 :(2)

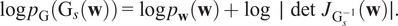



The second term can be dropped as the path length penalty in StyleGAN2 implies that the Jacobian determinant is constant for all 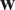 . The first term 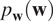 is the image prior we define on 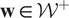 by(3)

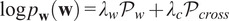

where

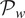
 is a prior that keeps 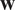 in the area of high density in 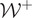 : 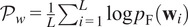 , where 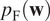 is estimated by a normalizing flow model 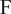 explained in further detail below.

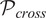
 is a pairwise Euclidean distance prior on 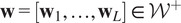 that ensures 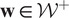 remains close to the trained manifold in 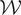 : 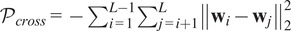 _._

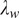
 and 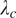 are hyperparameters that control the relative importance of the two priors.

#### 3.1.2. Normalizing flow

Using a sequence of invertible mappings, a normalizing flow 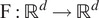 is a transformation of an unknown complex distribution into a simple probability distribution that is easy to sample from and whose density is easy to evaluate such as standard Gaussian.^(^^19^ ^)^

Let 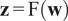 with probability density function 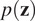 . Using the change-of-variable formula, we can express the log density of 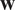 by^(^^20^ ^)^(4)

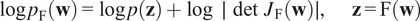

where 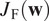 is the Jacobian of 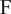 evaluated at 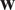 . In practice, the Jacobian determinant in equation nf should be easy to compute so that the density 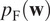 can be evaluated. Furthermore, as a generative model, the invertibility of 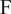 allows new samples 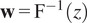 to be drawn through sampling from the base distribution. In the literature, several flow models were proposed, such as real non-volume preserving flow^(^^21^ ^)^ and masked auto-regressive flow (MAF).^(^^22^ ^)^

#### 3.1.3. Optimization process

In the likelihood term presented in equation map estimation, we assume that the noise follows a Laplace distribution, that is, 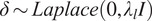 . This assumption simplifies the log density of 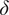 to 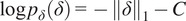 , where 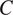 is a constant. Thus, the optimization problem in equation map estimation is effectively transformed into an optimization over 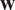 , resulting in the final objective function:(5)

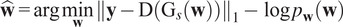



The selection of the Laplace distribution for noise modeling is favored due to its simple form, which simplifies the log-density function and makes the loss function easier to optimize. Moreover, the 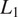 norm, which arises in the log density of the Laplace distribution, offers greater robustness to outliers than the 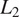 norm. This characteristic is particularly beneficial in the context of SR, where the ability to handle irregular noise or artifacts in LR images is crucial, especially given the challenges commonly associated with microscopy data.

### 3.2. Evaluation of the Gaussianization process

**Figure 1.**
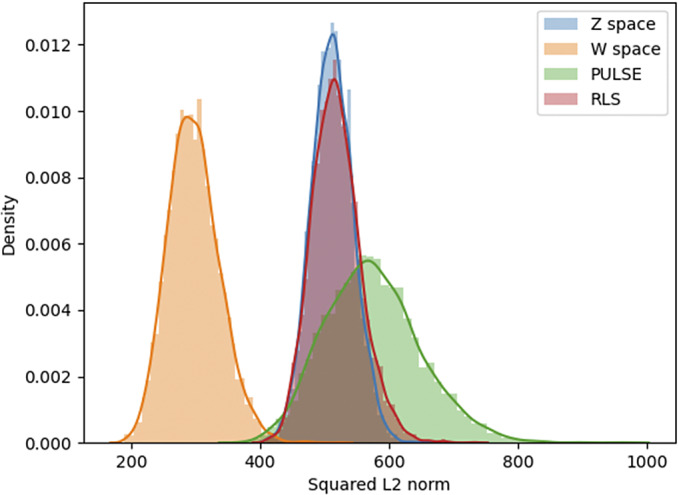
Distribution analysis of squared 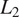 norms demonstrating the Gaussianization of latent style vectors. The graph compares the density of squared norms from the original 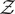 space (blue), the untransformed 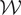 space (orange), and the distributions resulting from the PULSE (green) and our method (red).

To assess the quality of the Gaussianization process, we sampled 5000 vectors 
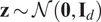
 . These vectors were then transformed into style vectors 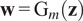 using the mapping network 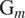 of StyleGAN. We then Gaussianized the distribution of 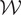 using alternatively PULSE and our method. The quality of the Gaussianization process was evaluated by computing the squared norm for all of these vectors within the transformed distribution (see Figure 1). Ideally, the squared norm of the standard Gaussian distribution 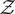 should follow a chi-squared distribution, that is, 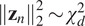 _,_ and thus, it forms a narrow distribution centered around 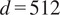 , the dimensionality of 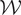 . We can see that this is the case for 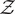 _,_ which is Gaussian. However, the 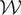 does not follow this pattern, which is inconsistent with the prior assumption held by BRGM (Bayesian Reconstruction through Generative Models).^(^^18^ ^)^ Furthermore, while the PULSE method results in a broader distribution, our method more accurately narrows the squared norm distribution to match the expected chi-squared distribution.

## 4. Experiments

### 4.1. Experimental settings

A critical aspect of our study was the necessity to work with high downscaling factors, such as 16x and 32x, which are central to our application scenario. These factors are not commonly used in the literature, and to the best of our knowledge, there are no experimental datasets available with such high downscaling factors. Most existing datasets typically focus on moderate downscaling factors of 2x or 4x, which is not aligned with the needs of our study, where we aim to address more extreme cases of resolution enhancement. Therefore, we had to generate our own LR images.

As for implementation details, we began by training a StyleGAN2-ADA (StyleGAN2 with Adaptive Discriminator Augmentation)^(^^23^ ^)^ model on a subset of the BBBC021 dataset,^(^^24^ ^,^^25^ ^)^ which comprises cells treated with drugs acquired following the cell painting assay.^(^^26^ ^)^ That is, cells were fluorescently stained with markers for F-actin, B-tubulin, and deoxyribonucleic acid (DNA), as described in.^(^^26^ ^)^ The dataset comprises wide-field epi-fluorescence images, captured using the automated ImageXpress imaging platform. From this dataset, we extracted images of size 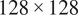 centered around each cell nucleus and used 400 images per compound treatment (approximately 28,000 in total) to train StyleGAN2-ADA.^(^^23^ ^)^ We then proceeded to generate 100000 random samples 
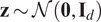
 and used their associated style vector 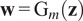 to train the normalizing flow model. We opted for MAF, as it tends to perform better than RealNVP (Real-valued Non-Volume Preserving) for density estimation tasks. Our normalizing flow model comprised five flow blocks with all hidden dimensions set to 1024. For super-resolving the images using our RLS algorithm, we employed an Adam optimizer over 200 iterations with a learning rate of 0.5 and initialized the search using the mean of 10,000 randomly generated latent vectors.

To fine-tune the regularization parameters 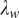 and 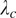 , we empirically evaluated a spectrum of values, eventually setting them to 5e-5 and 0.01, respectively. This process revealed that the algorithm’s performance remained relatively stable across a broad range of these parameters, indicating a lack of sensitivity as long as the values fell within a specific boundary. Precisely, we observed optimal performance when 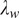 was between 1e-6 and 5e-4, and 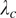 ranged from 0.005 to 0.05. These findings suggest that while the exact values of 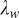 and 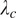 are flexible, maintaining them within these determined ranges ensures the algorithm functions effectively.

For evaluation, we used 100 random samples from various compound treatments, ensuring samples were not included in the training of the generative network. We simulated degraded images from these HR images using bicubic downsampling, which is a common choice in the literature. We then compared the results of our algorithm with those of Pix2Pix and two state-of-the-art unsupervised image reconstruction methods based on StyleGAN inversion: PULSE^(^^15^ ^)^ and BRGM.^(^^18^ ^)^ For the baseline methods, we used the same parameter settings reported by the original papers.

### 4.2. Results

#### 4.2.1. RLS recovers high-quality cell images

**Figure 2.**
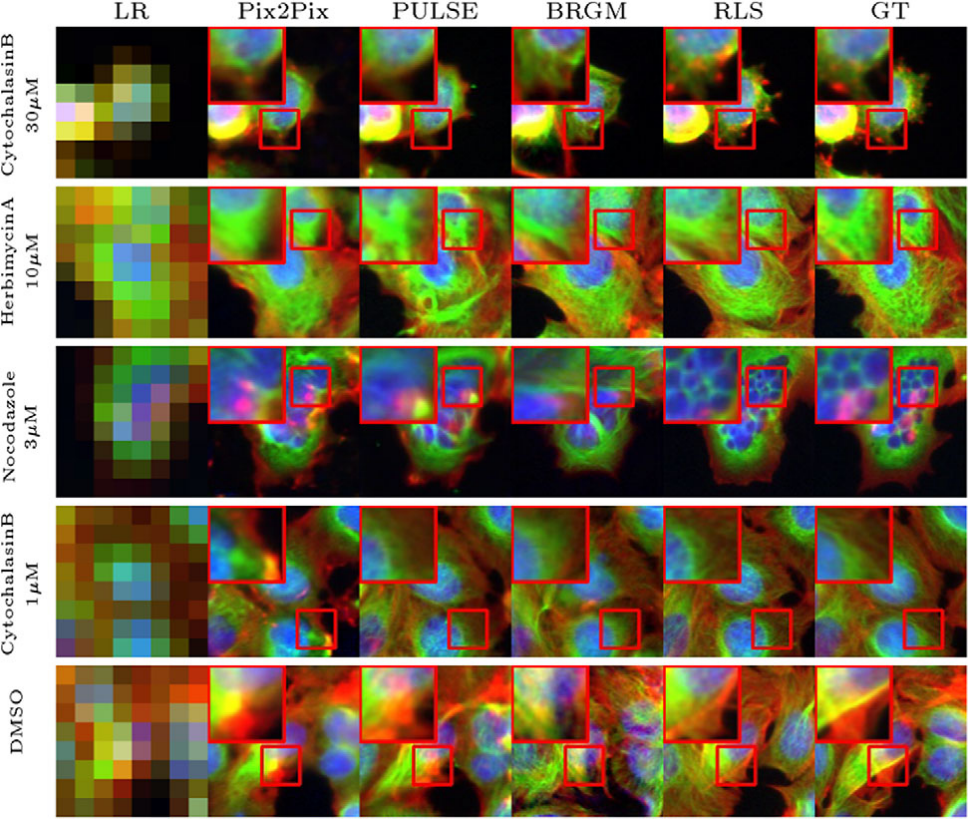
Qualitative comparison of SR reconstructions on the BBBC021 dataset: visualizing the performance of RLS against baseline methods in reconstructing cellular structures and phenotypes under negative control (DMSO) and various treatment conditions at a 16x upscaling factor.

We reconstructed images using RLS and compared them to images generated with baseline methods. The results of this comparison are presented in Figure 2. At these high upscaling factors, due to the lack of proper regularization, the competing methods fail to produce reasonable cellular details and tend to produce images that can be accurately downscaled to the LR image at the cost of generating distorted cellular structures. In contrast, our approach produces highly realistic images for most examples and can reproduce cell phenotypes induced by compound treatment even if the fine-grain details are not similar. For instance, when reconstructing the images of cells treated with nocodazole, a known microtubule destabilizer, RLS captures the typically fragmented microtubule phenotype, resulting in a more accurate texture. Reconstructing images of cells at higher resolution obviously provides access to finer-grain measurable features.

To quantitatively assess this gain in performance, we evaluate the SR images using Frechet inception distance (FID)^(^^27^ ^)^ and kernel inception distance (KID)^(^^28^ ^)^ to measure the discrepancy between the real HR images and the reconstructed one. As expected, the scores of FID and KID listed in Table 1 show that with both upscaling factors, our method significantly improves realism.

**Table 1.** Quantitative evaluation of RLS and baseline methods for SR on the BBBC021 dataset at 32x and 16x upscaling factors

Upscaling factor	Method	FID 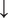	KID 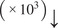	MSSIM 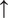	LPIPS 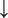	PSNR 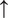
32x	Pix2Pix^(^^29^ ^)^	67.141	66.515	0.507	0.420	15.846
	PULSE^(^^15^ ^)^	*13.629*	*9.056*	*0.526*	*0.378*	*16.469*
	BRGM^(^^18^ ^)^	15.862	13.293	0.400	0.475	14.207
	RLS	**7.571**	**3.209**	**0.528**	**0.343**	**16.536**
16x	Pix2Pix^(^^29^ ^)^	45.503	41.700	*0.708*	0.274	*19.983*
	PULSE^(^^15^ ^)^	*17.354*	*14.937*	**0.723**	**0.244**	**20.806**
	BRGM^(^^18^ ^)^	19.862	17.254	0.608	0.301	17.914
	RLS	**6.983**	**3.347**	0.660	*0.262*	19.177

*Note*: The **best** and the second best are highlighted in bold and italic, respectively.

#### 4.2.2. RLS achieves better perceptual scores

We evaluated the reconstruction accuracy of RLS using learned perceptual image patch similarity (LPIPS),^(^^30^ ^)^ peak signal-to-noise ratio (PSNR), and multi-scale structural similarity index for motion detection (MS-SSIM)^(^^31^ ^)^ metrics to compare the reconstructed SR images to the real HR images. As expected, RLS did not achieve the best pixel-wise reconstruction losses but achieved either the lowest or second-lowest LPIPS scores on both 32x and 16x upscaling factors. The latter suggested that while the recovered SR image is not exactly the same as the HR image, it is perceptually closer. It is worth noting that although Pix2Pix produced images with high PSNR and MSSIM scores, it struggled to accurately reconstruct cellular details. This observation highlights the fact that PSNR and SSIM metrics may not be fully appropriate to evaluate the performances of the SR tasks.

#### 4.2.3. RLS preserves interpretable features

RLS achieves a balance between realism and fidelity when super-resolving microscopy images. However, as any deep learning method for SR, it cannot generate details it would not have seen during training. Therefore, it cannot be used in a context where novel events can be expected. However, it could possibly be used to perform measurements to quantify expected phenotypic changes. This is the case for many assays used in basic research in biology. It is also the case in diagnostics such as parasitemia for instance where the tool must assess if a parasite is present or not. Instead of relying on a blind classification or regression of LR images, these tasks on LR images in such a context could be decomposed into two steps. A first step based on our deep learning approach would consist of reconstructing a HR image, while a second step would use a dedicated handcrafted analysis to quantify a phenotypic feature, making the analysis explicitly interpretable.

To evaluate to what degree the information conveyed by the SR images can be used for such quantitative assays and maybe later for interpretable diagnostics, we applied it to two assays that allowed straightforward quantification of “interpretable features” on HR images but not on LR images. Here, interpretable features refer to the measurable and explainable phenotypic changes crucial for biological assessment, such as the nucleocytoplasmic ratio in response to TNF- 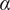 treatment and alterations in the Golgi apparatus morphology in reaction to nocodazole. Quantitative analysis was conducted on the SR images to assess the reconstruction of these features, and results were compared against those obtained from HR images as well as baseline methods, including BRGM, PULSE, and an unregularized latent search (“w/o Regu.”).

**Figure 3.**
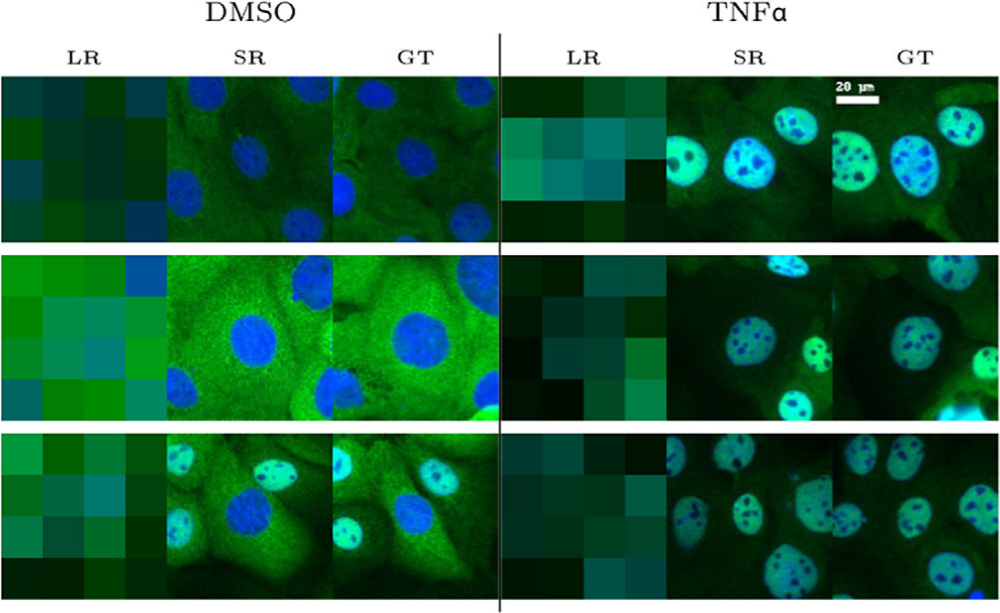
Visual examples of super-resolving translocation assay LR images at a 32x upscaling factor under negative control (DMSO) and tumor necrosis factor (TNF)- 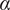 treatment conditions: left: LR image, middle: SR reconstruction, and right: GT.

The first assay aimed to track the location of the nuclear factor kappa B (NF- 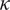 B) protein within the cell. Upon treatment with TNF- 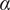 , a pro-inflammatory cytokine, the protein moves to the nucleus, causing a shift in fluorescence signal from the cytoplasm to the nuclear area, resulting in bright green nuclei. We observed that the nucleocytoplasmic fluorescence ratio measured on 1000 HR-treated and HR-untreated images could be replicated when computed from super-resolution (SR) images (Figure 5a). We also provided some visual examples of super-resolving the LR images, *that is*, the first step in Figure 3, which shows that our method can reconstruct images of wild-type and treated cell images obtained with this common assay.

**Figure 4.**
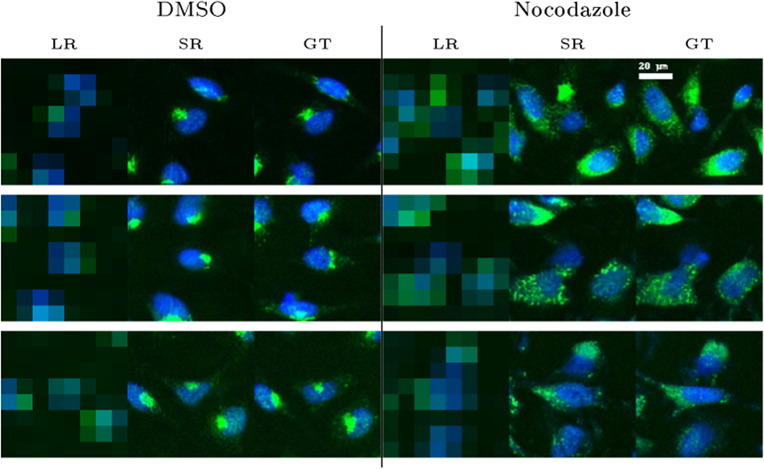
SR of images from a Golgi assay at a 16x upscaling factor under negative control (DMSO) and nocodazole treatment conditions. The left column shows the LR images, the middle column shows the SR reconstructions, and the right column shows the GT images.

**Figure 5.**
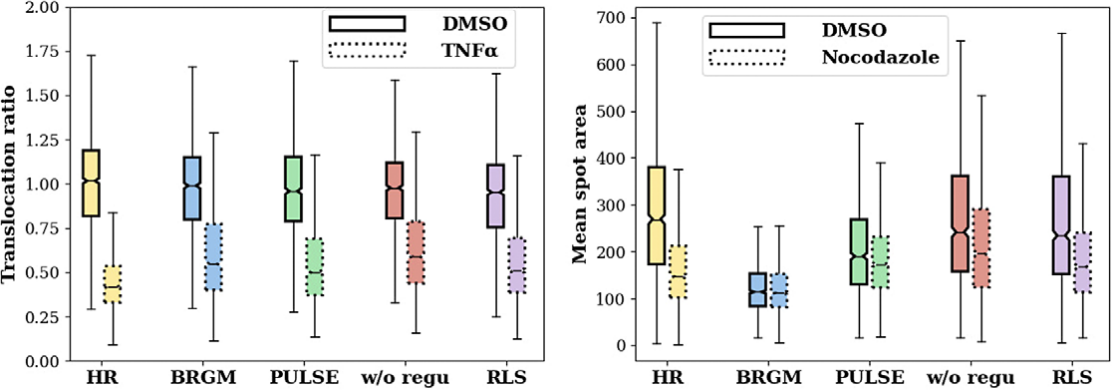
Making interpretable measurements from LR images: first increasing the resolution by a SR method and then measuring a handcrafted interpretable feature. Each pair in the boxplots displays the distribution of handcrafted interpretable measurements, where the solid box represents the negative control (DMSO) and the dotted box signifies the positive controls (TNF- 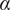 for translocation and nocodazole for Golgi), across various SR methods including RLS, BRGM, PULSE, and “w/o Regu.” alongside with the HR images for benchmarking. (a) Translocation ratio measurement: The y-axis quantifies the translocation ratio, an interpretable metric indicating TNF-induced NF- 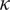 B translocation (green). The translocation ratio can be differentiated between two conditions not only from real HR images but also from SR images. (b) Mean spot area measurement: The y-axis quantifies the mean spot area, an interpretable metric indicating nocodazole-induced Golgi spreading (green), distinguishable between two conditions not only from real HR images but also from SR images reconstructed by our method.

The second assay we conducted aimed to monitor changes in the morphology of the Golgi apparatus. When cells are exposed to nocodazole, microtubules disassemble, causing the Golgi, originally located near the center of the cell, to break up into smaller stacks. Figure 4 shows that standard assays such as nocodazole-induced Golgi scattering (green) were reproduced with SR images. Moreover, as illustrated in Figure 5b, when computed only from SR images, a straightforward average spot size difference measured on 1000 HR-treated and 1000 HR-untreated images could be retrieved.

Furthermore, we utilized cells treated with DMSO (Dimethyl Sulfoxide) as negative controls and cells treated with nocodazole (or TNF in the case of the translocation assay) as positive controls. We developed a classifier trained on HR images to distinguish between the two phenotypes and evaluated its performance on both HR and super-resolved images, ensuring that the datasets for GAN training and classifier training were distinct to maintain the integrity of our results. Additionally, we trained a separate classifier on LR images for phenotype differentiation, and its testing was confined to LR images. The results as shown in Table 2 indicate that the SR operation does not degrade the discriminative signal contained in LR images, and the classification accuracy is about the same on SR and LR images. Furthermore, we show evidence that this signal difference can be retrieved when quantifying the same interpretable feature variation present in HR images using handcrafted image analysis (see Figure 5).

**Table 2.** Comparison of classification accuracy for identifying phenotypic changes between negative control (DMSO) and positive control (TNF- 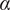 and nocodazole and nocodazole conditions) in the translocation and Golgi assays, respectively, using super-resolved images and HR images as a benchmark

	LR	BRGM	PULSE	w/o Regu.	RLS	HR
DMSO vs TNF– 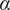 (32x)	85.60	79.20	85.60	85.80	87.00	97.60
DMSO vs nocodazole (16x)	72.80	53.00	62.60	67.80	74.20	96.80

It is worth noting that in Figure 5 the similarity in performance between the “w/o Regu.” model and RLS during the translocation assay reveals an interesting insight. It suggests that, for this specific assay, the additional regularization does not significantly change the outcome. However, it is important to note that this assay was designed to evaluate strong phenotypic changes where the expected changes are substantial and easy to detect. In this case, the nonregulated version can also accurately capture the phenotypic changes, since the differences between the images are large. This is evidenced by the comparative classification accuracies in Table 2 for the translocation assay, where RLS achieves similar performance to the “w/o Regu.” version and the baseline methods. The advantages of regularization in RLS are more apparent in assays with subtler phenotypic variations, where detecting nuanced differences is more challenging. For example, in the Golgi morphology assay, where detecting changes requires more detailed analysis, RLS’s regularization reconstructs realistic images that more faithfully represent biological structures required for detailed quantitative evaluations. Furthermore, considering the overall performance, RLS demonstrates consistent and enhanced performance compared to both the baselines and the non-regularized version, as indicated by the superior classification results in Table 2. This suggests that RLS is a more robust method for super-resolving microscopy images, especially in assays where the phenotypic changes are subtle. Overall, the experiments confirm that the quality of super-resolved images is adequate for further analysis. Our method enables a two-stage process: employing deep learning for the challenging task of SR followed by a handcrafted, interpretable method for the subsequent quantitative measurements.

### 4.3. Robustness

**Figure 6.**
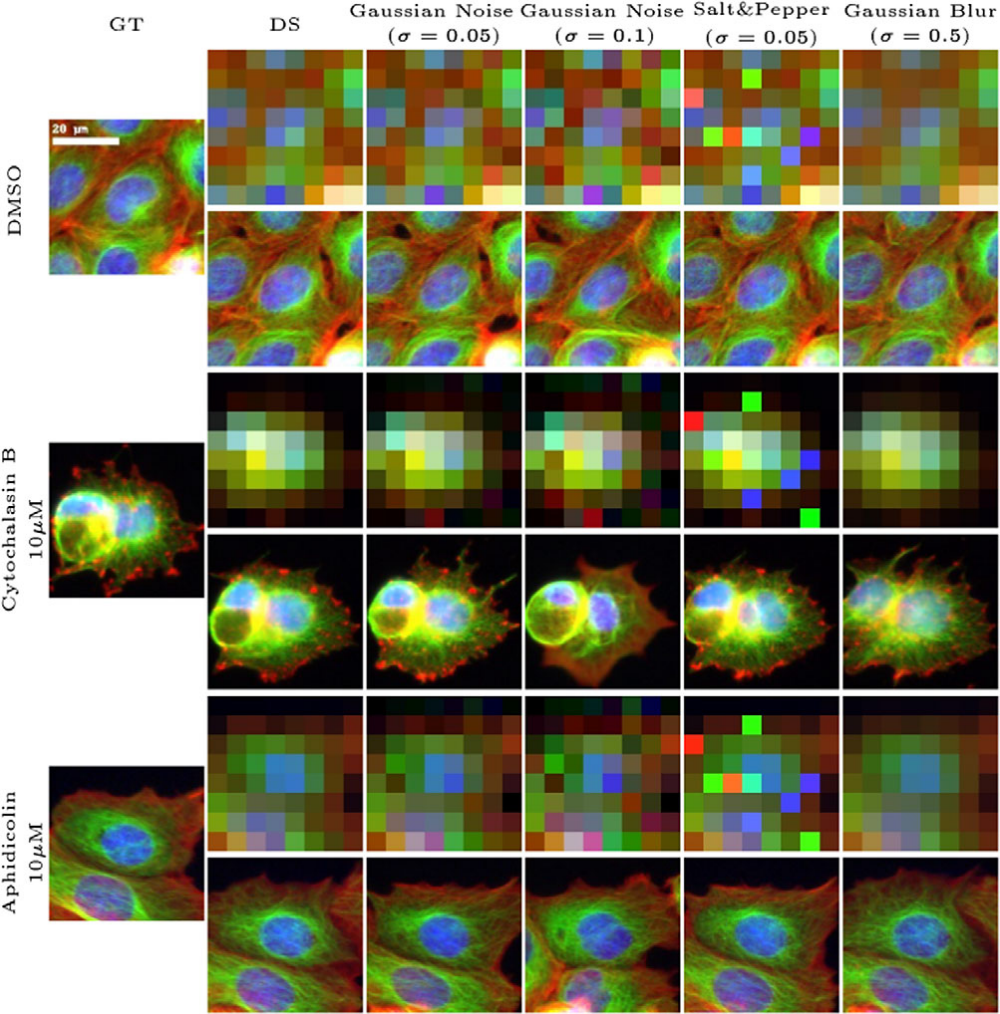
Evaluation of RLS performance under various degradation conditions: Images downscaled by bicubic method are further altered with Gaussian noise, salt and pepper noise, and Gaussian blur to assess the stability of the proposed method across a range of image perturbations (at a 16x upscaling factor).

In opposition to supervised methods that are sensitive to the input image domain, this approach is not restricted to a particular degradation operator that is used during training. To evaluate this aspect, we applied additional degradation operators such as Gaussian noise, salt and pepper, and Gaussian blur to a bicubic downscaled image DS before reconstruction. As depicted in Figure 6, the reconstruction closely matches the DS image. This result validates our choice of using the bicubic downscaling operator during training instead of more complicated specific degradation.

### 4.4. Ablation study

**Figure 7.**
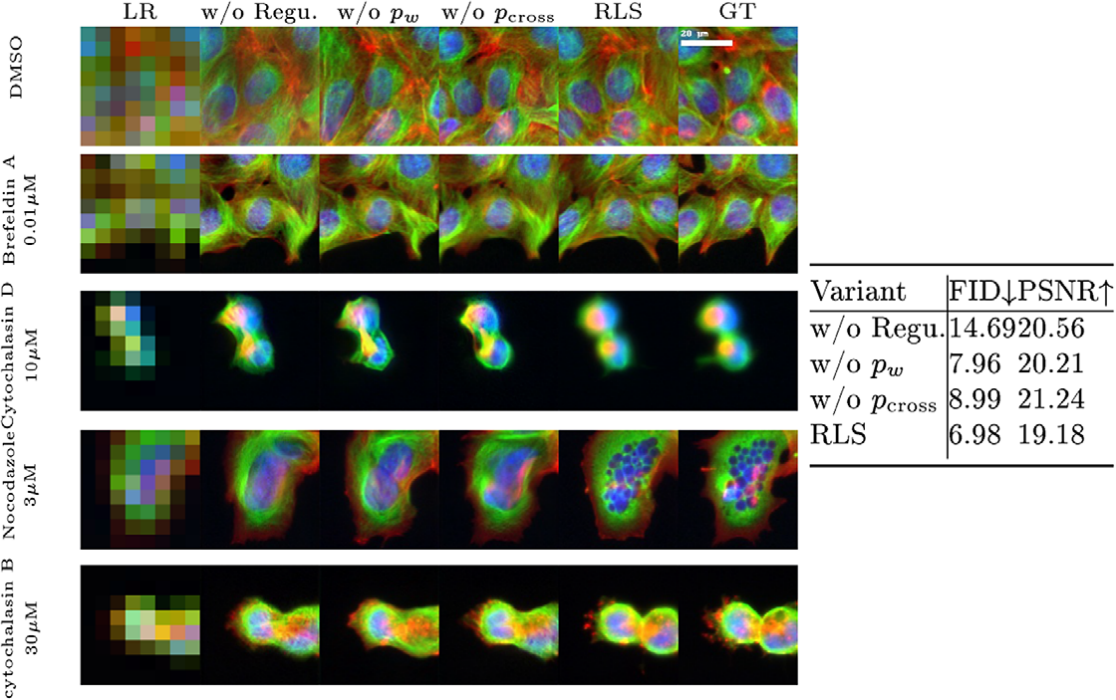
Ablation study showcasing the impact of regularization components on RLS performance, with qualitative results in the left column and quantitative results in the right column. The variants include “w/o Regu.” (searching the latent space without any regularization), “w/o 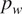 ” (the image prior does not include the prior term 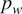 ), “w/o 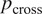 ” (the image prior does not include the prior term 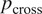 ), and “RLS” (the full RLS model) (at a 16x upscaling factor).

Figure 7 demonstrates ablation experiments highlighting the impact of the components of our image prior. First, “w/o Regu.” searches the latent space without any regularization for the image that, once downscaled, matches the LR image. The second variant is denoted “w/o 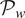 _,_” that is, the image prior does not include the prior term 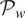 . Similarly, “w/o 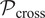 ” refers to the suppression of 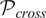 .

To evaluate the three variants, we use the same set of parameters described in Section 4.1. One can see that searching the latent space without any regularization produces images that do not necessarily belong to the original image manifold and therefore do not appear realistic. It also tends to generate images that are accurately downscaled to the LR image but at the cost of generating distorted images when 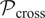 or 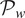 is discarded. This implies that both priors play an important role in generating realistic details.

### 4.5. Uncertainty

An important challenge of the SR task is that it is an ill-posed problem. Although we can improve this aspect by using an image prior constraint, several closely related highly resolved images could still be consistent with a single LR image. To generate 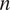 realistic SR images, we sample 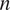 latent codes denoted as 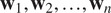 . We assume that their distribution follows a Gaussian distribution 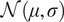 , where the parameter 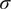 follows an inverse gamma distribution. Using the Bayes rule, we estimate the distribution’s parameters by

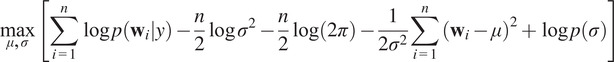



**Figure 8.**
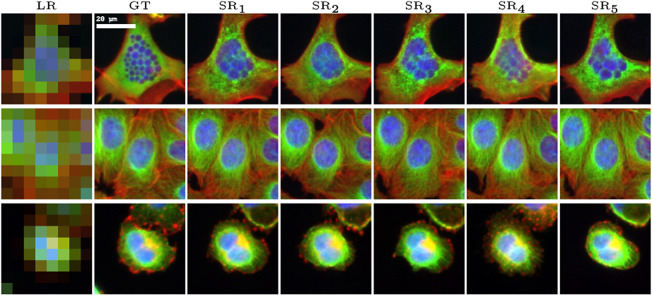
Visualizing the ill-posed nature of the SR task and the uncertainty associated with SR reconstruction. Five distinct SR images (SR_1_ to SR_5_) are generated for each LR image by sampling five different latent codes from the latent space, alongside the GT (at a 16x upscaling factor).

Here, the first term is the log-likelihood of the posterior distribution of 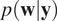 , which is defined in Eq. (5); the second term is the regularization term, which penalizes large values of 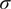 _;_ and 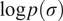 is the log prior distribution of 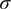 . Figure 8 displays multiple solutions for a given LR image we can obtain with our approach. Sampling close variations of HR images from a single LR input can be used to enhance the robustness of the estimation of an image-based quantitative feature by reducing the effects of noise or artifacts that may exist in the input LR image.

## 5. Conclusion

In this paper, we propose a robust SR method based on RLS within a pretrained StyleGAN. It does not require coupled image pairs for training and constrains the SR task to a given image prior to offering a trade-off between fidelity and realism of the SR reconstruction. Furthermore, we show that such a method could be used to split analyses, such as the classification of LR images, into a reconstruction of SR images performed by deep learning and a simple dedicated handcrafted analysis of an interpretable feature.

The latter could be used, for instance, for rapid diagnostic based on smartphone directly available on the field. In this case, a dataset of LR images of slides acquired from fast and/or cheap solutions available on the field could be coupled with the acquisition of the same slides using high-end expensive HR microscopes with limited access. In this application, HR images could then be reconstructed directly on the field to perform an explainable diagnostic such as a parasite count.

## 6. Limitations and future work

One common challenge with deep learning-based methods, especially those using generative priors like in our study, is their limited generalizability to unfamiliar and unseen data. Our approach, which utilizes StyleGAN for unsupervised learning within a specific domain, may not encompass the full diversity encountered in real-world scenarios. To enhance the model’s generalizability, strategies such as incorporating data augmentation to introduce training data variability, or applying transfer learning for domain adaptation, could be beneficial.

Our current findings serve as an initial validation of our method’s capabilities. However, we acknowledge the necessity of further evaluations using more varied datasets, encompassing a broader range of imaging techniques and sample preparation methods. The absence of such diverse experimental data in our current research is due to the specific requirement of our study to explore SR at high upscaling factors like 16x and 32x. These factors, which are crucial to our application’s needs, are rarely addressed in the available literature, and to our knowledge, datasets with such extreme upscaling factors are not yet available. Existing datasets generally focus on more moderate upscaling factors, such as 2x or 4x, which do not meet the demands of our research that targets significantly higher levels of resolution enhancement. We are committed to extending our validation to include real-world imaging conditions as soon as datasets meeting our high upscaling factor requirements become available. This will enable a more comprehensive assessment of our method’s applicability and performance in practical scenarios.

## Data Availability

We used the BBBC021 image set available from the Broad Bioimage Benchmark Collection (https://bbbc.broadinstitute.org/).

## References

[1] Weigert M, Schmidt U, Boothe T, et al. (2018) Content-aware image restoration: pushing the limits of fluorescence microscopy. Nature Methods 15(12), 1090–1097. 30478326 10.1038/s41592-018-0216-7

[2] Wang H, Rivenson Y, Jin Y, Wei Z, Gao R, Günaydın H, Bentolila LA, Kural C and Ozcan A (2019) Deep learning enables cross-modality super-resolution in fluorescence microscopy. Nature Methods 16(1), 103–110. 30559434 10.1038/s41592-018-0239-0 PMC7276094

[3] Ouyang W, Aristov A, Lelek M, Hao X and Zimmer C (2018) Deep learning massively accelerates super-resolution localization microscopy. Nature Biotechnology 36(5), 460–468. 10.1038/nbt.4106 29658943

[4] Hoffman DP, Slavitt I and Fitzpatrick CA (2021) The promise and peril of deep learning in microscopy. Nature Methods 18(2), 131–132. 33479523 10.1038/s41592-020-01035-w

[5] Gong Y and Sbalzarini IF (2015) A natural-scene gradient distribution prior and its application in light-microscopy image processing. IEEE Journal of Selected Topics in Signal Processing 10(1), 99–114.

[6] Zhu J-Y, Park T, Isola P and Efros AA (2017) Unpaired image-to-image translation using cycle-consistent adversarial networks. In Proceedings of the IEEE/CVF International Conference on Computer Vision. Venice, Italy: IEEE, pp. 2223–2232.

[7] Hell SW and Wichmann J (1994) Breaking the diffraction resolution limit by stimulated emission: stimulated-emission-depletion fluorescence microscopy. Optics Letters 19(11), 780–782. 19844443 10.1364/ol.19.000780

[8] Betzig E, Patterson GH, Sougrat R, Lindwasser OW, Olenych S, Bonifacino JS, Davidson MW, Lippincott-Schwartz J and Hess HF (2006) Imaging intracellular fluorescent proteins at nanometer resolution. Science 313(5793), 1642–1645. 16902090 10.1126/science.1127344

[9] Nehme E, Weiss LE, Michaeli T and Shechtman Y (2018) Deep-storm: super-resolution single-molecule microscopy by deep learning. Optica 5(4), 458–464.

[10] Karras T, Laine S and Aila T (2019) A style-based generator architecture for generative adversarial networks. In Proceedings of the IEEE/CVF Conference on Computer Vision and Pattern Recognition. Long Beach, CA, USA: IEEE, pp. 4401–4410. 10.1109/TPAMI.2020.2970919 32012000

[11] Karras T, Laine S, Aittala M, Hellsten J, Lehtinen J and Aila T (2020) Analyzing and improving the image quality of stylegan. In Proceedings of the IEEE/CVF Conference on Computer Vision and Pattern Recognition. Seattle, WA, USA: IEEE, pp. 8110–8119.

[12] Abdal R, Qin Y and Wonka P (2019) Image2stylegan: How to embed images into the stylegan latent space? In Proceedings of the IEEE/CVF International Conference on Computer Vision. Seoul, South Korea: IEEE, pp. 4432–4441.

[13] Wulff J and Torralba A (2020) Improving inversion and generation diversity in stylegan using a gaussianized latent space. arXiv preprint arXiv:2009.06529.

[14] Bora A, Jalal A, Price E and Dimakis AG (2017) Compressed sensing using generative models. In Proceedings of the International Conference on Machine Learning. Sydney, Australia: PMLR, pp. 537–546.

[15] Menon S, Damian A, Hu S, Ravi N and Rudin C (2020) Pulse: Self-supervised photo upsampling via latent space exploration of generative models. In Proceedings of the IEEE/CVF Conference on Computer Vision and Pattern Recognition. Seattle, WA, USA: IEEE, pp. 2437–2445.

[16] Zhu P, Abdal R, Qin Y, Femiani J and Wonka P (2020) Improved stylegan embedding: Where are the good latents? arXiv preprint arXiv:2012.09036.

[17] Goodfellow I, Bengio Y and Courville A (2016) Deep Learning. Cambridge, MA, USA: MIT Press.

[18] Marinescu R, Moyer D and Golland P (2021) Bayesian image reconstruction using deep generative models. In NeurIPS Workshop on Deep Generative Models and Downstream Applications. Online: NeurIPS.

[19] Kobyzev I, Prince SJD and Brubaker MA (2020) Normalizing flows: An introduction and review of current methods. 43(11):3964–3979. 10.1109/TPAMI.2020.2992934 32396070

[20] Papamakarios G, Nalisnick ET, Rezende DJ, Mohamed S and Lakshminarayanan B (2021) Normalizing flows for probabilistic modeling and inference. 22(57), 1–64.

[21] Dinh L, Sohl-Dickstein J and Bengio S (2016) Density estimation using real nvp. arXiv preprint arXiv:1605.08803.

[22] Papamakarios G, Pavlakou T and Murray I (2017) Masked autoregressive flow for density estimation. In Proceedings of the Advances in Neural Information Processing Systems. Long Beach, CA, USA: Curran Associates, Inc., pp. 2338–2347.

[23] Karras T, Aittala M, Hellsten J, Laine S, Lehtinen J and Aila T (2020) Training generative adversarial networks with limited data. In Advances in Neural Information Processing Systems. Red Hook, NY, USA: Curran Associates, Inc., pp. 12104–12114.

[24] Caicedo JC, McQuin C, Goodman A, Singh S and Carpenter AE (2018) Weakly supervised learning of single-cell feature embeddings. In Proceedings of the IEEE/CVF Conference on Computer Vision and Pattern Recognition. Salt Lake City, UT, USA: IEEE, pp. 9309–9318. 10.1109/CVPR.2018.00970 PMC6432648 30918435

[25] Ljosa V, Caie PD, Ter Horst R, et al. (2013) Comparison of methods for image-based profiling of cellular morphological responses to small-molecule treatment. Journal of Biomolecular Screening 18(10), 1321–1329. 24045582 10.1177/1087057113503553 PMC3884769

[26] Caie PD, Walls RE, Ingleston-Orme A, Daya S, Houslay T, Eagle R, Roberts ME and Carragher NO (2010) High-content phenotypic profiling of drug response signatures across distinct cancer cells phenotypic profiling across cancer cell types. Molecular Cancer Therapeutics 9(6), 1913–1926. 20530715 10.1158/1535-7163.MCT-09-1148

[27] Heusel M, Ramsauer H, Unterthiner T, Nessler B and Hochreiter S (2017) Gans trained by a two time-scale update rule converge to a local nash equilibrium. In Advances in Neural Information Processing Systems. Red Hook, NY, USA: Curran Associates, Inc., pp. 6626–6637.

[28] Bińkowski M, Sutherland DJ, Arbel M and Gretton A (2018) Demystifying mmd gans. arXiv preprint arXiv:1801.01401.

[29] Lee H-C, Cherng ST, Miotto R and Dudley JT (2019) Enhancing high-content imaging for studying microtubule networks at large-scale. In Proceedings of Machine Learning for Healthcare Conference. Ann Arbor, MI, USA: PMLR, pp. 592–613.

[30] Zhang R, Isola P, Efros AA, Shechtman E and Wang O (2018) The unreasonable effectiveness of deep features as a perceptual metric. In Proceedings of the IEEE/CVF Conference on Computer Vision and Pattern Recognition. Salt Lake City, UT, USA: IEEE, pp. 586–595.

[31] Wang Z, Simoncelli EP and Bovik AC (2003) Multiscale structural similarity for image quality assessment. In Proceedings of Asilomar Conference on Signals, Systems & Computers, Vol. 2, pp. 1398–1402. Pacific Grove, CA, USA: IEEE.

